# Promising Novel Therapies in the Treatment of Aortic and Visceral Aneurysms

**DOI:** 10.3390/jcm12185878

**Published:** 2023-09-10

**Authors:** Theodora M. Stougiannou, Konstantinos C. Christodoulou, Efstratios Georgakarakos, Dimitrios Mikroulis, Dimos Karangelis

**Affiliations:** Department of Cardiothoracic Surgery, University General Hospital of Alexandroupolis, Dragana, 68100 Alexandroupolis, Greece; konstantinoschristodoulou@yahoo.gr (K.C.C.); efstratiosgeorg@gmail.com (E.G.); dmikrou@med.duth.gr (D.M.); dimoskaragel@yahoo.gr (D.K.)

**Keywords:** cardiothoracic surgery, vascular surgery, tissue engineering, mesenchymal stem, miRNA, cell therapy, vascular biology, regenerative medicine, matrix metalloproteinase inhibitors

## Abstract

Aortic and visceral aneurysms affect large arterial vessels, including the thoracic and abdominal aorta, as well as visceral arterial branches, such as the splenic, hepatic, and mesenteric arteries, respectively. Although these clinical entities have not been equally researched, it seems that they might share certain common pathophysiological changes and molecular mechanisms. The yet limited published data, with regard to newly designed, novel therapies, could serve as a nidus for the evaluation and potential implementation of such treatments in large artery aneurysms. In both animal models and clinical trials, various novel treatments have been employed in an attempt to not only reduce the complications of the already implemented modalities, through manufacturing of more durable materials, but also to regenerate or replace affected tissues themselves. Cellular populations like stem and differentiated vascular cell types, large diameter tissue-engineered vascular grafts (TEVGs), and various molecules and biological factors that might target aspects of the pathophysiological process, including cell-adhesion stabilizers, metalloproteinase inhibitors, and miRNAs, could potentially contribute significantly to the treatment of these types of aneurysms. In this narrative review, we sought to collect and present relevant evidence in the literature, in an effort to unveil promising biological therapies, possibly applicable to the treatment of aortic aneurysms, both thoracic and abdominal, as well as visceral aneurysms.

## 1. Introduction

From the second half of the 20th century, many technological advances have breathed new life into the fields of molecular biology and biotechnology. Novel biological resources like stem and progenitor cells can now be deployed to generate and refine biomaterials, which could potentially tackle previously complicated and high-mortality clinical entities [1]. In particular, the application of these new technologies and biomaterials in cardiovascular diseases is potentially vast, from production of cell lineages for tissue replacement, to the fusion of cells with extracellular materials to develop ready-made tissues. It is evident that the target has now changed. The goal is no longer only to replace, but also to regenerate and reinstate the tissue, when possible [2,3,4].

Studies delving into the pathophysiological state of vascular injury and how local progenitor cells contribute to the process, along with studies about vascular tissue development, have spurred the idea of vascular repair using progenitors. This became apparent since various stem populations have been found to contribute to vascular remodeling [5]. Research into the pathogenesis of aneurysmal disease, coupled with the comparison of the cellular arrangement between healthy and diseased vessel wall, has demonstrated the potential for application of stem and progenitor cells to mimic or augment physiological tissue regeneration [6].

On the other hand, well-known complications of the widely implemented in-surgical repair of aortic aneurysms vascular grafts, such as endoleaks, graft thrombosis, “compliance mismatch”, or complications related to endovascular ablation for visceral aneurysms [7], may jeopardize the post-operative outcome. More suitable materials with bioactive properties, that better match or even imitate native tissues, capable of addressing the biomolecular alterations seen with aneurysm pathology have emerged as new treatment options. Hence, we sought to perform a narrative review of the literature, to unveil promising biological therapies, possibly applicable to aortic as well as visceral artery aneurysms (VAA).

## 2. Definition and Epidemiology of Aneurysms

According to the 2014 European Society of Cardiology and the corresponding guidelines published in 2022 by the American College of Cardiology, a uniform definition for aneurysms does not exist. Descending thoracic aortic (TAA) and abdominal aortic aneurysms (AAA) are defined as any dilatation of at least 1.5 times the initial diameter of the affected vessel. Regarding the ascending aorta, an increase in diameter greater than 4.0 cm is considered a dilatation, while an increase greater than 4.5 cm is defined as an aneurysm [8].

With an estimated incidence of 5.9 per 100,000 person-year, which is constantly increasing [9], a thoracoabdominal aneurysm (TAAA) is defined as an aortic aneurysm that may extend into both the thoracic and the abdominal aorta. TAAAs may be categorized according to the Crawford Classification into five types, which in general, describe aneurysm morphology and aid in patient risk stratification [9,10].

On the other hand, visceral artery aneurysms (VAA), i.e., aneurysms affecting various visceral arteries, including the splenic, the hepatic and the mesenteric arteries, have a population incidence of about 0.01% to 0.1%, and are thus considered relatively rare [11]. Furthermore, within this cohort, splenic artery aneurysms seem to be the most frequent, comprising 60% of the cases [11].

## 3. Etiology of Aortic and Visceral Aneurysms: Genetic Syndromes vs. Sporadic Disease

Typically, aortic aneurysms occur sporadically. Tobacco abuse, uncontrolled hypertension, older age, and even an untreated chronic aortic dissection are considered the main predisposing factors [9,12]. Atherosclerosis, although more frequently implicated in descending thoracic and abdominal aortic aneurysms, might also be an etiologic factor for TAAAs and VAAs [13,14], while inflammatory disorders such as Takayasu arteritis, infectious aortic disease, or trauma may also be to blame [8]. Moreover, hemodynamic alterations, or even hormonal changes during pregnancy, in the case of splenic artery aneurysms for example, might be predisposing factors as well [11]. Aortic aneurysms have also been associated with various genetic disorders, including but not limited to Marfan Syndrome (MFS), type IV Ehlers-Danlos Syndrome (EDS) (vascular EDS) [15], autosomal dominant polycystic kidney disease (ADPCKD), and Turner Syndrome (XO). Many of these syndromes have been linked with both thoracic and abdominal aortic diseases [16,17,18,19,20,21]. Moreover, vascular Ehlers-Danlos syndrome seems to be responsible for genetic predisposition to VAA occurrence as well [22].

## 4. Symptomatology of Aortic and Visceral Aneurysms

Though initially asymptomatic, as the aneurysm progresses, pressure on adjacent tissues might cause symptoms in about 57% of patients prior to rupture, including hoarseness and dysphagia due to compression of the left laryngeal nerve or esophagus, respectively, as well as gastrointestinal obstruction due to impingement on the nearby small intestine [23]. TAAAs in particular, may be also the cause of vague and chronic chest or abdominal pain, with signs of erosion typically presenting as hemoptysis or hematemesis, whereas VAAs, though also usually asymptomatic, might present with intraperitoneal hemorrhage and bleeding from the portal venous system [24]. In addition, retrograde expansion of some TAAAs, as well as thoracic aortic aneurysms (TAAs), might lead to dilation of the aortic annulus, causing aortic valve regurgitation and aortic dissection [25].

## 5. Molecular Pathophysiology of Aortic and Visceral Aneurysms

Aneurysms generally involve derangement of normally occurring molecular processes in the aortic wall. In general, every cell type within this microenvironment may be affected and contribute alone or together with others towards aneurysm formation and progression. Endothelial cells (ECs) may contribute to aneurysmal pathogenesis through derangement in the expression of cell adhesion molecules, or expression of pro-inflammatory cytokines, including TNF-a. These molecules may lead to increased leukocyte adhesion, oxidative stress, and inflammation within the wall [26], causing endothelial injury. This is further supported by relevant studies in animal models of TAA, which have shown that when intercellular EC adhesion molecules, such as tight junctions (TJs) and adherens junctions (AJs), are preserved, no TAA formation is observed [27]. It therefore seems that inflammation indeed plays a role at this stage of pathogenesis. Furthermore, as it is a characteristic finding in AAA, it could be also be implied in TAAA formation [9], while it is implicated in VAAs pathogenesis as well, especially celiac artery and mesenteric artery aneurysms [28,29]. ECs, in this case, may often display a pathological phenotypic change, termed epithelial to mesenchymal transition (EpMT), which includes loss of cellular adhesion characteristics, and acquisition of a mesenchymal phenotype, along with increased protein secretion, tendency for migration, and leukocyte adhesion molecule expression; this further disrupts the endothelial barrier, augmenting circulating leukocyte recruitment, and as a result, inflammation [30,31].

Another important factor is disruption of the normal extracellular matrix ECM turnover; increased matrix metalloproteinase (MMP) activation leads to increased collagen and elastin hydrolysis [32,33], in turn causing unopposed ECM degradation. Two specific metalloproteinases have been found to be implicated; MMP9 has been associated with aneurysmal wall dilatation [34], while MMP2 has been identified in higher quantities in disease-free segments. Furthermore, altered expression of tissue inhibitor of metalloproteinases (TIMPs) [35] has also been linked to disease pathogenesis, as shown in relevant animal models.

Phenotypic derangement of vascular smooth muscle cells (VSMCs) and their subsequent apoptosis [33,36] might also be a contributing factor; though a normal equilibrium between synthetic (sVSMC) and contractile VSMCs (cVSMCs) populations does normally exist, inflammatory conditions in the aortic wall have been shown to favor VSMC phenotype cycling towards a more proliferative state, leading to an alteration in aortic contractile properties. These proliferative VSMCs are also abnormal, since they have been shown to express both contractile and synthetic protein markers, and are more susceptible to apoptosis as well, eventually causing thinning of the tunica media [31]. It thus seems that progressive weakening of the medial layer, which can sometimes be attributed to an alteration in flow and hemodynamic conditions, as is the case with splenic artery aneurysms for example [29,37], is central to the development of both aortic aneurysms and VAAs [38].

A number of intracellular and extracellular signaling pathways have been found to be associated with aneurysm development in the aorta; one is the MAPK/ERK pathway, which, through sequential kinase activation, triggers NF-Kβ expression [39], although impairment of the AKT2 pathway may also contribute to aneurysm formation [40,41]. Disrupted TGF-b signaling may also be implicated in pathogenesis, due to possible alterations in VSMC and EC phenotype, causing these cells to acquire mesenchymal characteristics; eventually, loss of the normal cell phenotype occurs, with disturbance of the endothelial barrier, and eventual leukocyte infiltration within the aortic wall [31]. In addition, TGF-b signaling may affect the relative quantities of extracellular fibrillar proteins and cellular cytoskeleton organization, thus affecting cellular movement. Finally, altered TGFB receptor functions may affect the MAPK pathway as well [42,43].

**Figure 1:**
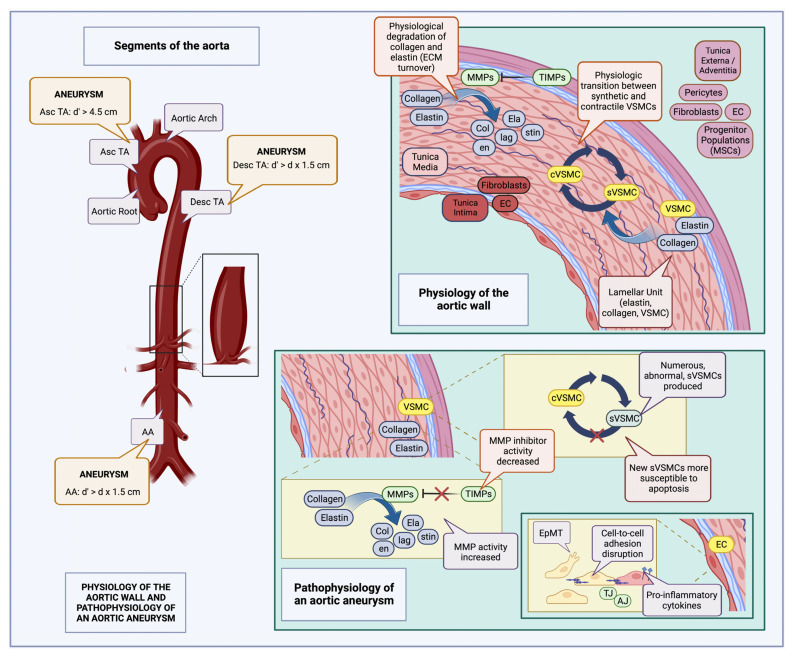
Physiology of the aortic wall and pathophysiology of an aortic aneurysm (created with BioRender.com): The aorta is composed of three layers, the tunica intima containing ECs along with fibroblasts in the subendothelial layer, the tunica media, composed of elastin interlaced with collagen, and VSMCs (lamellar units), and the tunica externa (adventitia), composed of pericytes, fibroblasts, ECs, and various progenitor cell populations, such as MSCs. Normally, there is a constant turnover of collagen and elastin fibers in the tunica media, carried out by MMPs and inhibited by TIMPs; there is also an equilibrium between synthetic (sVSMC) and contractile VSMC (cVSMC) populations [8,44,45]. Aneurysm definitions may vary according to location; thus, for the Asc TA, it is defined as a diameter (d’) exceeding 4.5 cm, while for the Desc TA, AA, as well as many visceral vessels, it is defined as an increase in diameter greater than the product of the initial vessel diameter multiplied by 1.5 (d’ > d x1.5) [8]. During aneurysm development, many of these normal physiological processes are disrupted. Abnormal sVSMCs, more susceptible to apoptosis, accumulate [36], there is increased MMP activity along with associated decreased TIMP activity, leading to augmented fragmentation of collagen and elastin fibers. In addition, EC intercellular adhesion is interrupted, due to AJ and TJ derangements, as well as switching of the EC phenotype, from an epithelial to a mesenchymal type; pro-inflammatory cytokines are also expressed on the EC surface, facilitating leukocyte entry into the aortic wall [41,46,47]. MSC: mesenchymal stem cells; TIMP: tissue inhibitors of metalloproteinase; MMP: matrix metalloproteinase; EC: endothelial cell; VSMC: vascular smooth muscle cell; TJ: tight junctions; AJ: adherens junctions; EpMT: epithelial-to-mesenchymal transition; Asc TA: ascending thoracic aorta; Desc TA: descending thoracic aorta; AA: abdominal aorta; d: initial aortic diameter; and d’: aneurysmal aortic diameter.

Shiz Aoki; Katya Shteyn; and Ryan Marien; Figure 1: Physiology of the aortic wall and pathophysiology of an aortic aneurysm—created with BioRender.com. Available online: https://www.biorender.com/about (accessed on 18 July 2023).

## 6. Animal Models of Aneurysm

As with any new therapeutic intervention, prior to clinical implementation, appropriate preclinical tests both in vitro as well as animal studies are warranted. Usually, each animal model utilizes a specific molecular alteration contributing to aneurysmal pathophysiology. Some, for example, utilize infusion of a metalloproteinase, or any other type of protease enzyme to mimic ECM degradation. To this end, the enzyme elastase can be infused within the aortic lumen or added to the adventitia [48,49]. From these two options, the less invasive adventitia model can also be utilized for TAA, though full recapitulation of events observed in the latter cannot be achieved through use of these aforementioned elastase models alone [50,51].

Other effective models are chemically induced, such as the CaCl_2_ model and the various decellularized xenograft models. Adding calcium chloride onto the adventitial layer of the aorta promotes calcification and degradation of elastin in the tunica media; furthermore, it can mimic both AAA and TAA [52,53]. Xenograft models generally utilize aortic specimens derived from different species, after decellularization and implantation onto a different host animal; the ensuing immune reaction causes degradation of ECM components [54], thus mimicking aspects of aortic aneurysm pathogenesis.

## 7. Current Treatment Strategies

### 7.1. Conservative Management

Aneurysms may be addressed conservatively with medical therapy and serial observation/follow-up, or they may be treated surgically. Conservative treatment generally aims to reduce growth rates, aneurysm-related mortality risk, and cardiovascular events, as well as ensuring a favorable post-operative outcome when used in conjunction with operative treatment [8,55]. Certain lifestyle modifications such as tobacco cessation and mild physical exercise may also have a positive effect on patients’ overall health [56,57,58]. On the other hand, while there is a scarcity of data regarding physical activity and dietary alterations, both are routinely recommended [55]. It is also worth noting here, the paradox associated with diabetes mellitus (DM) having a protective effect on aortic wall homeostasis; this might be due to modulation of factors associated with inflammation during states of hyperglycemia, though the exact pathophysiologic mechanisms have not been yet fully explored [59].

In order to alleviate the inflammation and associated shear stress on the aortic wall, blood pressure and cardiac contractility should be regulated [8,55]. Since uncontrolled hypertension poses a silent threat, which could lead to aortic dissection or even rupture, a blood pressure target of 130/80 mmHg is highly endorsed, though occasionally, a lower cut-off (<120 mmHg) is set if tolerated [60]. Finally, to further diminish the rate of potential cardiovascular adverse events and remodeling, as well as hinder aneurysm growth, statin uptake is also suggested [61].

Over time, with progressive dilation of the aneurysmal sac, an intraluminal thrombus may form [55]. The presence, and most importantly, the size of the thrombus, have both been implicated in further aneurysm progression and rupture; as such, initiation of an antithrombotic-antiplatelet regimen has been proposed [62,63], recommended for aortic aneurysms and also applicable to VAAs, such as renal artery aneurysms [63,64].

### 7.2. Criteria and Forms of Surgical Intervention

Repair in TAAs is usually indicated when the diameter reaches a size equal to or greater than 5.5 cm. However, in patients with genetic disease or other concomitant risk factors, intervention may be warranted at lower thresholds, for example, at 5 cm [8]. For AAAs on the other hand, repair is generally decided at a threshold equal to or greater than 5.5 cm for men and 5.0 cm for women [8].

Indications for surgical intervention in TAAA are not as clear cut as for AAAs, mostly due to a lack of appropriate, high-level evidence. In general, the presence or risk of rupture and acute dissection, the size of the aneurysm (diameter ≥ 6.0 cm, or ≥5.5 cm if carried out at specialized, multidisciplinary aortic centers), rapid growth > 1 cm, a penetrating atherosclerotic ulcer [8], and symptoms such as aortic valve insufficiency (when the ascending aorta is affected) are the main indications [9,65].

With regard to VAA treatment, for some, surgical repair is warranted once a certain diametric threshold is overcome if no other symptoms are present (for example, ≥3 cm for splenic artery aneurysms and ≥2 cm for celiac and jejunoileal artery aneurysms). For other VAA types, including pseudoaneurysms of any kind, as well as superior mesenteric (SMA) and colic artery aneurysms, surgical repair is usually indicated once diagnosis is confirmed [64].

Though open repair has been the most commonly utilized method for aortic and visceral aneurysm repair, it does not come without complications. Whereas massive hemorrhage, cardiac arrest, and multisystem organ failure might lead to death in extreme scenarios, there can also be debilitating complications that increase post-operative patient morbidity, such as paraplegia due to spinal cord ischemia and renal failure [66]. The advent of new techniques utilized in open repair however have considerably improved survival and reduced some of the associated post-operative complications [67,68]. An article published in 2022 elucidated the instances where open or endovascular repair might be favored, respectively. According to this, low-risk patients with unfavorable anatomy, chronic dissection, genetic disease, and appropriate cardiopulmonary reserve may be treated with open surgical repair. High-risk patients on the other hand, or patients with favorable aortic anatomy, may be appropriately tackled with endovascular methods [69,70].

Total endovascular repair (TEVAR) is an endovascular option for surgical treatment in both aortic aneurysms and VAAs [7,64]; endovascular devices used for this purpose may be multibranched, fenestrated, or both. The latter technique, termed FB-EVAR, i.e., fenestrated branched endovascular repair [71], when employed in the repair of aortic aneurysms, comprises a safe alternative method [72]. Though endovascular repair, compared to conventional open surgery, does exhibit better results in terms of morbidity and mortality, its potential complications warrant diligent attention and may demand reoperation. Apart from the possibility of endoleak, the stent itself may migrate, collapse, or become infected (in select cases of graft infection, mortality rates reach 50%), and its limbs may kink or occlude completely. There may also be systemic complications, including limb, renal, or other visceral organ ischemia as well as pelvic ischemia [73].

## 8. Promising New Updates

### 8.1. A Discovery of Stem Cells: Stem Cell Therapies for Aneurysms

For the purposes of this review, cell therapies are defined as all those therapeutic interventions that utilize some type of cell to tackle the pathophysiologic alterations observed in aortic and visceral aneurysms. Tissue engineering might also utilize stem or progenitor cells during vascular graft generation, possibly leading to convergence between different therapies; indeed, many tissue-engineered vascular conduits might be seeded with stem cells or their products, during their development. In this section however, therapies involving the direct use of cells will be examined, or in other words, cells delivered in a way that does not constitute an organized vascular tube.

#### 8.1.1. Stem and Progenitor Cells: Definitions, Classification, and Derivation

Stem cells (SC) are defined as cellular populations existing in an undifferentiated or partially differentiated state, capable of generating differentiated daughter cells (potency) as well as replenishing their own populations (self-renewal) [74]. They may be classified based on their potential for the generation of differentiated progeny into totipotent stem cells (TSC), pluripotent stem cells (PSC), and multipotent stem cells; totipotency refers to the capability of a particular cell to generate an entire organism, including all embryonic, germline, and extraembryonic tissues [75,76]. Pluripotent stem cells (PSC) on the other hand, are capable of generating all three embryonic cell lineages; human embryonic stem cells (hESCs), a subset of PSCs, are usually generated from the inner cell mass (ICM) of developing blastocysts [77,78], while induced pluripotent stem cells (iPSC) can be generated from any type of cell through application of a cocktail of transcription factors, as first elucidated by Takahashi et al. as well as Yu et al., with the combinations OCT-4, SOX-2, NANOG, and LIN23 (OSNL) [79], and OCT-4, C-MYC, SOX-2, and KLF-4 (OMSK) [80,81,82,83,84,85]. Finally, multipotent stem cells usually produce differentiated cell types from the specific organ or tissue they inhabit; for example, MSCs found in various tissues, can generate cells found in adipose, osteogenic, and connective tissue [86].

#### 8.1.2. Cell Therapies for Aneurysms

Regarding cell therapies applicable to aneurysms, it seems that multipotent stem cell types such as MSCs have been used in experimental studies of aortic aneurysm [87], though studies with non-stem cell therapies have also been published [88], with these evaluated in AAA models [87].

Various animal studies have been conducted to test MSC effects in AAA pathology, which themselves have been further assessed through meta-analysis; the studies compared however were heterogenous [87]. Some of the primary outcomes evaluated were aortic diameter, amount of elastin within the aortic wall, expression of inflammatory mediators, as well as enzyme activity. Concerning aortic diameter, Li et al. [87] showed that adipose tissue—derived and allogeneic MSCs—had the greatest effect [89,90], which may be attributed to the greater compatibility between cells of the same species, as opposed to interspecies MSC–organism interactions. Effects have also been reported on elastin concentration, MCP-1, TNF-a, IL-6, and other inflammatory cytokines, as well as MMP activity; specifically, MMP concentration seemed to be reduced, while TIMP activity was increased [87]. MSCs have been administered either directly around the area of interest, (perivascularly [91], within its lumen [92,93]), or intravenously; once again, evaluation of relevant experimental studies through meta-analysis by Li et al. has shown that perivascular incubation or localized intraluminal administration produced the greatest effect [87].

MSCs might be preferred due to their immunomodulatory properties, as well as their ease of derivation, since they can be obtained not only from bone marrow (BM), but from adipose tissue specimens as well. The main effect of MSC therapy might be derived not as much from generation of appropriate differentiated progeny, but from the anti-inflammatory, immunomodulatory effects, thus it is a therapy of paracrine effect [94].

More terminally differentiated cell types might also have a place in the treatment of aneurysm. For example, ECs have been utilized in a rat xenograft model, to evaluate their efficacy in stabilization of the aortic intimal lining. The results were promising, with prevention of aneurysm formation measured through aortic diameter expansion rates as well as recruitment of VSMC and subsequent ECM generation [95]. Another type of cell therapy worth noting is VSMC administration; VSMCs are normally found within the tunica media, and their numbers are usually decreased in aneurysms. Allaire et al., applying VSMCs intraluminally in a rat xenograft aortic model, showed that 8 weeks after infusion, inflammatory cell infiltration was decreased, along with matrix MMP activity, while TIMP activity was increased, possibly through tunica media stabilization [96].

### 8.2. Tissue Engineering (TE)

Tissue engineering (TE), in general, is defined as a process that aims to generate functional and compatible tissue replacement constructs. For this purpose, appropriate biomaterials, which may or may not be organized into distinct scaffold shapes, as well as cells and other signaling molecules or growth factors, are used [97,98]. TE applications for vascular replacement usually revolve around the use of appropriate vascular scaffolds, which may or not be seeded with cells before implantation. The biomaterials used must exhibit similar mechanical and, when possible, similar biodegradability and biological activity to native tissues [99]; biodegradability allows for gradual integration of the implanted construct onto surrounding tissues, facilitating its eventual replacement by host cells and tissues [100,101].

Various fabrication methods may be used to generate vascular scaffolds, either through removal of cells from a preexisting biological shape, known as decellularization, or through ground-up construction from their constituent parts. Decellularization, for example, uses appropriate chemical solutions to strip the cellular components of preexisting vascular tissue, which may be later seeded with patient-specific cells [102]. Phase separation, electrospinning, and self-assembly may also be utilized; the two former methods generally utilize a tubular, rotating mandrel and either take advantage of the differential solubility of the component polymers or high voltage, to drive the polymer solution towards a collector, eventually creating a tubular structure. Self-assembly, on the other hand, involves use of cell populations, which secrete appropriate extracellular material that can then be arranged into a scaffold structure, appropriate for vascular tissue engineering (TE) [103,104].

#### Tissue Engineered Vascular Grafts (TEVGs) and Large Vessel Replacement

Engineering large diameter vessels, with the appropriate cellular composition and histological organization, is a mechanically challenging process. Many studies employing TEVGs in large animal models have been carried out, while other studies have assessed the use of TEVG for larger vessel replacement in isolated clinical cases or patient groups; some of these will be presented in this section.

Any TEVG capable of replacing a large caliber vessel, including aortic and visceral vessels, must have several characteristics. Firstly, it must possess appropriate biocompatibility, which depends both on the composition of the biomaterial itself, as well as its surface, to avoid thrombus formation. The luminal surface of a scaffold may be coated with hydrophilic synthetic polymers, such as polyethylene glycol (PEG) and heparin. However, to prevent overt hydrophilicity of the generated intimal surface, binding peptides such as RGD, CAG, REDV, and YIGSR, specifically recognized by endothelial cells (ECs), may be added so that EC adhesion and formation of an endothelial layer may occur appropriately within the scaffold (endothelialization) [105].

In addition, if a separate cell seeding stage is employed during fabrication, cells must be distributed to their appropriate locations as evenly as possible, to properly mimic the native vessel. Proper cell distribution might be challenging, as cellular composition in vascular tissue is characterized by the presence of layers. As such, cell seeding stages are often insufficient, though methods to utilize the cell adhesion properties of fibronectin have been used to promote cell adhesion within the graft [106].

A particularly challenging aspect in the creation of large diameter vessels is the utilization of appropriate components and maturation methods that allow for mechanical strength. The final product should be mechanically stable, possessing enough elasticity to properly mimic the native vessel, withstand the high pressures developed in the larger vessels, and allow for appropriate surgical manipulation [107]; to this end, after a vascular construct is generated, maturation must take place within a bioreactor, to create a final product appropriate for further use [108].

To generate a large diameter vessel, cells along with an appropriate scaffold must be utilized. In order to avoid immune rejection, autologous cells are often preferred, however, the harvesting of various types of differentiated vascular cells is cumbersome and cannot be used to acquire cells in an appropriate amount. The advent of stem cell biology techniques has aided with the harvesting process; stem or progenitor cells from the patient may be acquired and propagated in culture. Both pluripotent and multipotent stem cells may be used to generate the required cell populations in vitro. While multipotent stem cells may be used shortly after isolation, their limited numbers and the fact that their quality may be affected by the age and physiological state of each patient make their acquisition technically harder [109]. On the other hand, the generation of the appropriate cell populations from patient-derived iPSCs might be easier to accomplish since the latter may be produced from any cell. Thus, VSMCs, ECs, and other cell types capable of populating the vascular scaffold may be produced from such iPSCs; the tumorigenic potential of some of the transcription factors used to generate iPSCs however might hamper any perceived excitement. Though more research on the tumorigenicity of iPSC-derived cells might be needed, promising results were shown by Luo et al. as no cancer was documented in subjects after transplantation of the hiPSC-infused TEVGs [110].

The mechanical requirements of the biomaterial scaffold construct are of the utmost importance in large diameter vessels, as is the case with the aorta and the visceral vessels. While traditional synthetic textiles like Dacron and ePTFE are used for aortic grafts, their biological profile, potential for mimicking native biological tissue, and the possibility for compliance mismatch might limit their utility [111]. Thus, for TE applications, relevant textiles have been modified through the addition of biologically active substances. Bhattacharva et al. tested the use of Dacron ^®^ (PET) grafts with embedded CD34+ bone marrow cells on canine models, to test endothelialization in target grafts as well as formation of microvessels, which indeed seemed to occur. Still, the graft had a mostly synthetic, non-biodegradable scaffold, not permitting for the appropriate integration and replacement by natural tissues [112].

Hoerstrup et al., in their longitudinal study, focused on synthetic biodegradable scaffolds, testing large diameter TEVGs generated from PGA (poly-glycolic acid) and seeded with lamb fibroblasts and ECs, as a replacement for pulmonary arteries. While the study sample consisted only of 14 subjects, the up to 100-week follow-up showed that the replacement vessels exhibited a composition similar to native vessels, with appropriate functionality [113]. Another example of a biosynthetic, biodegradable large diameter tissue-engineered vessel was a PLCL-PGA (poly(L-lactide-co-ε-caprolactone)—poly-glycolic acid) construct seeded with bone marrow mononuclear cells (BM-MNC), as a connecting conduit between the inferior vena cava and the pulmonary artery in a 3-year-old female patient with congenital cardiovascular malformations. Following an interval of 11 years, the implanted TEVG was patent and capable of growth along with the host organism [114,115].

Some experimental studies have started using combinations of natural biodegradable polymers like agarose, alginate, or gelatin materials, seeded with SC populations, which can generate the appropriate differentiated progeny, while fabrication methods such as cell sheet rolling have also been used. An example of the latter includes the studies by L’Heureux et al. to generate a TEVG tested in a canine animal model. Though a short term study, with the TEVG lasting for about 6 days, the model exhibited appropriate mechanical strength and good surgical handling [116]; subsequent use of a similarly constructed TEVG in human patients for dialysis access [117] resulted in a 60% patency at 6 months, with some patients requiring further intervention for stenosis and occlusion, and one patient exhibiting dangerous hemorrhage [114]. Other studies have been using additive manufacturing or 3D printing to generate vascular tissue constructs based on the natural, biodegradable polymers previously mentioned; Gao et al., for example, generated a construct produced with alginate and human VSMC and ECs, later implanted as part of the rat aorta, as a proof-of-concept study, though it was not comparable in size to a human-size aorta [118].

Many of the large diameter vessel replacement studies have also been using animal scaffolds through decellularization procedures; in fact, decellularization as a process for TEVG production has been utilized for quite a few decades, with some animal-derived scaffolds already available commercially, such as the bovine carotid artery graft, Artegraft [114], which is said to be on par with ePTFE grafts in terms of quality [119]. It seems that decellularization techniques might be favorable due to an already present, mechanically, and biologically appropriate ECM configuration, which can be improved upon, and seeded with the appropriate cell populations. More specifically, laboratory studies have thus far utilized xenogeneic aortas from large animals to generate TEVGs seeded with cells; Bader et al., for example, utilized a decellularization protocol to preserve the ECM structure of porcine aortas, which were then later seeded with ECs and myofibroblasts. These were then transplanted into rat models to evaluate host tissue reaction and integration, with promising results [120]. Other studies, such as the study by Aldridge et al., aimed at evaluating the biological and mechanical characteristics of decellularized scaffolds for large vessel replacement; the study showed appropriate histological composition and biomechanical characteristics, after decellularization of donor human aortas, though no cells were added [121].

While scaffold fabrication and composition are of great importance, maturation under appropriate conditions is also required for the final product to possess appropriate biological and mechanical qualities for in vivo applications. To generate aortic conduits, bioreactors, which can mimic the physiological environment of large arterial vessels may be employed; thus, appropriate control of not only biological parameters important for cellular and histological maturation, but pressure and flow parameters as well, is essential for the conduit to mature under the appropriate conditions. Some studies, including the one conducted by Wang et al., utilized commercially available bioreactors [122], while others, such as the study carried out by Pennings et al. [123], crafted their own. Parameters that allow for better maturation of conduits may include pulsatile flow and appropriately high pressure, as well as physiological levels of shear stress; not only did these parameters aid in the better development of TEVG mechanical properties, but they allowed for better biological maturation of cells involved, thus improving overall tissue structure [124].

### 8.3. Biological Factors

Though use of growth factors and signaling molecules along with stem cells or during TEVG fabrication has already been explored, this section mostly examines use of standalone biological molecules or biological factors, and their potential for aortic and visceral aneurysm therapy.

The implications of EC function disruption in the pathogenesis of aortic aneurysms, along with their associated proteins, has already been explored. Dysfunction of various cell adhesion molecules can destabilize intimal continuity, favor adhesion of circulating leukocytes and lead to exposure of the aortic wall structure to inflammatory mediators leading to aortic wall injury. A study by Yang et al. examined the effect of AT-1001, or larazotide acetate in the attenuation of thoracic aortic aneurysm (TAA) and dissection in a mouse model. It was shown that AT-1001 preserved the distribution of proteins participating in cell–cell adhesion, such as ZO-1 and claudin-5, in both ascending and descending aortic sections. It thus functioned as a TJ sealing or stabilizing agent, preventing the accumulation of inflammatory mediators within the aortic wall, and therefore the advent of aneurysmal dilatation. AT-1001 as a synthetic peptide has already been tested in various inflammatory disorders, for example inflammatory bowel disease (IBD), and could thus be a useful adjunct for treatment of large vessel aneurysms as well [125].

ECM degradation in the tunica media is a well-known factor that promotes dilatation and aneurysm progression, associated with unopposed overexpression or overactivation of matrix metalloproteinases (MMP), and decreased activity of inhibitors of metalloproteinase (TIMP) function. Various studies have explored the effects of metalloproteinase inhibition on aortic aneurysm development. Several MMP inhibitors (MMPi) have been or are currently being studied/tested in clinical trials for treatment of AAA. These may mimic and share a protein structure required to bind the required MMP sites, known as peptidomimetic, such as the hydroxamic acid molecules batimastat and marimastat, which entered phase III clinical trials in 2020. Alternatively, they may only mimic MMPs without sharing a protein structure, and are thus termed non-peptide MMPis, such as disulfiram, which has been observed to possess antitumor effects and is also already in use for treatment of chronic alcohol intoxication, or a synthetic MMPi, known as XL784, both of which entered phase II clinical trials in 2020. Peptidomimetic MMPis target a variety of MMPs, including MMP-2, -3, -9; however, they have been associated with low bioavailability and musculoskeletal side effects, both of which may be tackled with nanoparticle delivery systems [126]. On the other hand, XL784 has been found to have high specificity for MMP-2, with less associated musculoskeletal symptoms [127].

Another type of molecule that has recently gained quite a bit of traction is miRNA or microRNA, which can affect gene and protein expression, either through miRNA–mRNA interaction, or through interaction with gene promoters. miRNAs may be delivered through exosomes, i.e., lipid vesicles usually derived from the cellular endosomal system [128], or bound to certain proteins, i.e., Argonautes [129]. It is also worth noting that miRNAs are extremely stable in extracellular fluids [130], which no doubt could further aid their possible delivery as biological therapy agents.

In vivo, miRNA imbalances may be implicated in the development of aortic aneurysms. In fact, miR-21 has been shown to be elevated in AAA animal models, though its effect has been mostly shown to be protective. miR-21 is normally associated with proliferation and apoptosis of VSMCs, acting through the AKT pathway; hence, augmentation of miR-21 numbers might aid in mitigating aneurysm dilatation and progression, since miR-21 antagonism in relevant experiments resulted in greatly enhanced aneurysm progression [131].

Additional studies in murine models of TAA by Akerman et al. revealed another miRNA crucial in aneurysm pathophysiology. miR-133a activity is normally associated with fibroblast function; as such, it can be inferred that during TAA progression, derangements in aortic fibroblasts, in part due to a decreased in miR-133a concentration, might also contribute to the general pathology. It was further shown, that in cases of miR-133a overexpression, TAA progression was halted [132]; miR-133a thus presents another interesting target for treatment of aneurysm.

All novel biological interventions presented in this text, applicable in aortic and visceral aneurysms, are summarized in Table 1 (Table 1).

**Figure 2:**
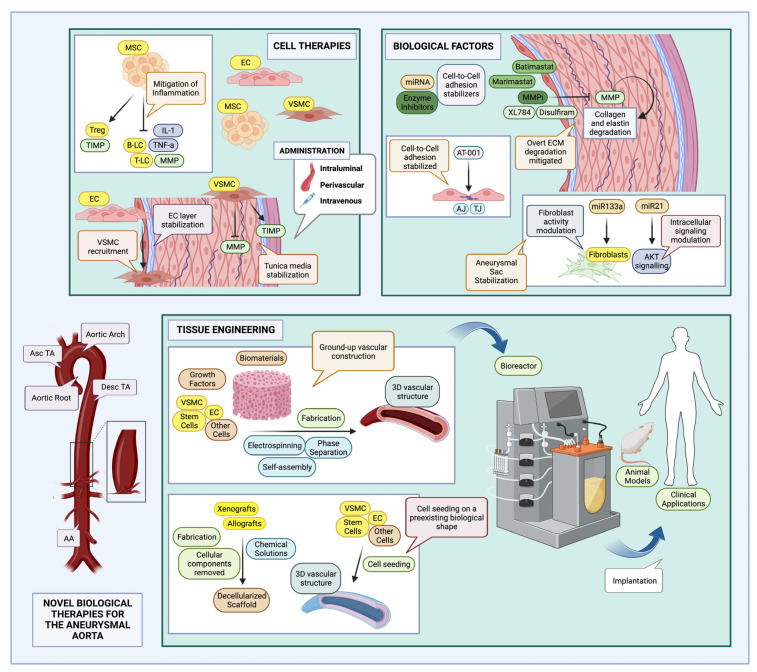
Novel biological therapies for the aneurysmal aorta (created with BioRender.com): These may take the form of cell therapies, tissue engineered vascular grafts (TEVGs), or various biological factors. Cell therapies can encompass both stem and non-stem cells, acting mainly in a paracrine fashion [87]. Therapies with TEVGs usually aim to replace the diseased aortic segment with a bioengineered alternative; various fabrication methods may be used, and constructs may be generated from the ground-up, or animal/human grafts may be decellularized with chemical solutions and populated with patient cells before implantation [108]. Biological factors may also be utilized to strengthen endothelial intercellular connections [125], inhibit MMP enzymes [126], or modulate cell function [131,132]. MSCs: mesenchymal stem cells; Treg: T regulatory lymphocytes; TIMPs: tissue inhibitors of metalloproteinases; B-LC: B-lymphocytes; T-LC: T-lymphocytes; IL-1: Interleukin-1; TNF-a: tissue necrosis factor—alpha; MMP: matrix metalloproteinase; EC: endothelial cell; VSMC: vascular smooth muscle cell; MMPi: matrix metalloproteinase inhibitors; TJ: tight junctions; AJ: adherens junctions; ECM: extracellular matrix; AKT: also known as protein kinase B (PKB); Asc TA: ascending thoracic aorta; Desc TA: descending thoracic aorta; AA: abdominal aorta; d: initial aortic diameter; and d’: aneurysmal aortic diameter.

Shiz Aoki; Katya Shteyn; and Ryan Marien; Figure 2: Novel biological therapies for the aneurysmal aorta—created with BioRender.com. Available online: https://www.biorender.com/about, (accessed on 18 July 2023).

## 9. Conclusions

From stem cells to cell-biomaterial combinations to biological factors such as proteins and miRNA, it seems that the possibilities of these biological therapies are indeed promising. The immunomodulatory capabilities of MSCs render them perhaps ideal candidates to tackle most of the biomolecular imbalances observed in aortic and visceral aneurysms, in the case of tackling elements of inflammation and immune cell activation. On the other hand, TEVGs pose a much more convoluted and potentially rewarding solution. Since aortic and large vessel artery repair in general entails the replacement of a diametrically large, arterial segment, grafts created through bioengineering methods for this purpose must withstand high mechanical and flow conditions, as well surgical handling, and suturing. Different methods have been used so far to create TEVGs, although most have been tested only in animal models, apart from some exceptions in specific patients, or small patient groups. Biological factors that target key processes in the pathophysiology of an aortic aneurysm have been already tested, such as EC cohesion factors or more recently, MMPis. miRNA therapy might also represent an interesting alternative; once again, it is by observation of physiological and pathological processes that certain miRNA molecules have been found to be critical in progression or mitigation of aneurysmal pathogenesis.

However, further research needs to be carried out in certain areas, to increase current knowledge. More specifically, while animal models of TAA and AAA exist, there is no animal model specifically geared towards recapitulating TAAA or VAA pathology. Though it stands to reason that some aspects of the pathophysiological process are shared, elucidating any possible, specific derangements in the normal physiology that might cause dilatation of such large segments in the aorta (in the case of TAAAs) or that might affect only specific visceral vessels is essential. With regard to cell therapies, more experimental studies evaluating either the potential for MSCs to tackle the proposed pathophysiological disturbances through generation of differentiated progeny or the potential for pluripotent stem cells to achieve vessel wall regeneration in a similar manner are required. Τwo methods this could be achieved by could be either through direct application of the aforementioned cell types and observation for differentiation events in vivo, or through derivation of differentiated vascular cell types from either MSCs or pluripotent stem cells.

In the case of TEVG studies, there are either a large variety of studies in different large animals or in isolated small patient group studies. It is important that studies are grouped based on the type of material and fabrication method and systematically compared to better understand the characteristics of TEVGs based on each different biomaterial combination used. The use of 3D printing is especially promising, and as such, more studies in large animal models testing different material combinations should be carried out to evaluate aspects such as immunogenicity and biomechanical characteristics. It is also important that relevant studies, either in animal models or patient groups, are conducted for long periods of time to better assess any change in material characteristics or possible material degeneration, as well the extent of assimilation by host tissues, through time.

Finally, with regard to use of individual biological factors, more data reporting the role of different miRNAs might be a useful adjunct to additional pathophysiology studies to uncover new molecular targets for the treatment of both aortic and visceral aneurysms. Though some MMPis are already under evaluation, it is important to assess whether there are any other existing pharmaceutical compounds, already in use for other conditions, as for example, is the case with disulfiram, which might have similarly beneficial effects.

## Figures and Tables

**Table 1:** Summary of new biological therapies applicable in aortic and visceral aneurysms.

Intervention	Target/Action	Application	Additional Information
**MSC**	MCP-1, TNF-a, IL-6, MMP/TIMP	Small animal models (AAA)	Meta-analysis of 18 studies in animal models, by Li et al. [87], showed significant effect on aortic diameter, inflammatory mediators, and elastin content; there were also effects on enzyme activity, with decreased MMP and increased TIMP expression levels.
**EC**	Tunica intima stabilization, VSMC recruitment	Small animal models (AAA)	Administration in a rat model by Franck et al. [95] prevented new AAA formation and expansion of preexisting AAAs.
			Observed effects by Franck et al. [95]: Indirect stabilization of the endothelium through paracrine effects, and augmentation of aortic wall components through ECM secretion via VSMC recruitment.
**VSMC**	Tunica media stabilization, MMP/TIMP	Small animal models (AAA)	Intraluminal use in a rat model by Allaire et al. [88,96]; decrease in inflammatory cell infiltrate, decreased MMP, and increased TIMP activity.
**TEVG**	Large vessel replacement	Canine model	Large artery synthetic graft (Dacron^®^) infused with CD34+ BMCs by Bhattacharya et al. [112]; successful endothelialization and microvessel formation; incomplete integration by host.
	Large vessel replacement	Large animal model (lamb)	Pulmonary artery replacement by Hoerstrup et al. [113]. Hoerstrup et al. [113] used large diameter TEVG fabricated from PGA scaffold, seeded with lamb ECs and fibroblasts; 14 subjects were only followed for 100 weeks, appropriately functioning graft during this time.
	Large vessel replacement	Patient with congenital heart malformation	Biosynthetic, biodegradable TEVG fabricated from PLCL-PGA scaffold, seeded with BM-MNCs in a 3-year-old female patient; appropriately functioning during a follow-up period of 11 years [114].
	Large vessel replacement	Canine model	Large vessel TEVG fabricated (L’Heureux et al. [116]) through VSMC and fibroblast sheet rolling, with EC seeding; short term implantation in canines (6 days), appropriate biomechanical characteristics and handling.
	Large vessel replacement	Renal dialysis patients	Large vessel TEVGs, also fabricated through cell-sheet rolling, exhibited 60% patency at 6 months, when transplanted in 6 patients (L’Heureux et al. [117]); additional interventions due to restenosis and occlusion were required; one patient exhibited dangerous hemorrhage [114].
	Small vessel replacement	Rat model	Biodegradable TEVG fabricated from natural polymer (alginate) containing human VSMCs, ECs through 3D printing; implanted as part of the rat aorta [118].
	Large vessel replacement	Artegraft	Decellularized bovine carotid artery graft, commercially available, described by some as having similar quality to ePTFE grafts [114,119].
	Experimental evaluation of immunogenicity	Rat model	Decellularized porcine aorta seeded with human ECs and myofibroblasts; immunogenicity evaluated through implantation in a rat model; exhibiting appropriate integration and host reaction was observed [120].
	Experimental evaluation of biomechanical characteristics	In vitro	Biomechanical characteristics of a decellularized human aorta were tested by Aldridge et al.; no cell seeding stages [121].
**AT-1001 (larazotide acetate)**	Cell–cell adhesion (ZO-1, Claudin-5)	Murine model (TAA)	TJ sealing agent (preserves distribution of cell-adhesion proteins); tested in both ascending and descending thoracic aortic sections (Yang et al. [125]) Also assessed for the treatment of inflammatory bowel disease (IBD) [125].
**Batimastat, Marimastat**	MMP (such as MMP-2, MMP-3, MMP-9)	Phase III Clinical Trials (2020) (AAA)	MMPi (peptidomimetic) with low bioavailability, musculoskeletal side effects; proposed nanoparticle delivery systems to improve side effects and bioavailability [126].
**Disulfiram**	MMP (MMP-2, MMP-9), Type IV collagenase	Phase II Clinical Trials (2020) (AAA)	MMPi (non-peptide) [126] Already in use, for treatment of chronic alcohol intoxication (FDA-approved) [126] Antitumor effects [126]
**XL784**	MMP (High specificity for MMP-2)	Murine Models (AAA) Phase II Clinical Trials (2020) (AAA)	MMPi (non-peptide, synthetic molecule); fewer musculoskeletal symptoms compared to peptidomimetic MMPis [127].
**miR-21**	AKT signaling pathway	Murine Models (AAA) Human aortic samples (AAA)	Augmentation mitigates aneurysm progression (interference with VSMC proliferation and apoptosis pathways); delivery through exosomes or bound to proteins (i.e., Argonautes) (Maegdefessel et al. [131]).
**miR-133a**	Fibroblast	Murine Models (TAA)	Overexpression halts aneurysm progression by affecting fibroblast function; delivery through exosomes or bound to proteins (i.e., Argonautes) (Akerman et al. [132]).

**Note**. This table is a summary of the information presented in Section 8 “Promising new updates”, noting the type of therapy applied, the target of each therapeutic intervention, the target application (clinical trials, animal models), and any additional information pertaining to each intervention. The table was created by the authors to summarize the new biological therapies already presented in the main text of the present paper. AAA: abdominal aortic aneurysm; AKT: also known as protein kinase B (PKB); EC: endothelial cell; BMC: bone marrow cells; PGA: polyglycolic acid; PLCL: poly(L-lactide-co-ε-caprolactone); ePTFE: expanded polytetrafluoroethylene; BM-MNC: bone marrow mononuclear cells; IL-6: interleukin-6; MCP-1: monocyte chemoattractant protein-1; MMP: matrix metalloproteinase; MSC: mesenchymal stem cells; TAA: thoracoabdominal aortic aneurysm; TEVG: Tissue-engineered vascular grafts; TIMP: tissue inhibitors of metalloproteinase; TNF-a: tumor necrosis factor alpha; TJ: tight junctions; VSMC: vascular smooth muscle cell; ZO-1: zonula occludens-1.

## Data Availability

Not applicable.
